# Effect of antioxidant supplements on sperm parameters in infertile male smokers: a single-blinded clinical trial

**DOI:** 10.3934/publichealth.2020009

**Published:** 2020-02-11

**Authors:** Safa Sadaghiani, Soghra Fallahi, Hossein Heshmati, Saeed Hosseini Teshnizi, Hooshang Abedini Chaijan, Fahimeh Farzad Amir Ebrahimi, Farhad Khorrami, Mina Poorrezaeian, Faezeh Alizadeh

**Affiliations:** 1 Department of Nursing, Faculty of Nursing and Midwifery, Hormozgan University of Medical Sciences, Bandar Abbas, Iran; 2 Fertility and Infertility Research Center, Hormozgan University of Medical Sciences, Bandar Abbas, Iran; 3 Social Determinants in Health Promotion Research Center, Hormozgan Health Institute, Hormozgan University of Medical Sciences, Bandar Abbas, Iran; 4 Hormozgan University of Medical Sciences, Bandar Abbas, Iran

**Keywords:** antioxidant, clinical trial, ROS, smokers, sperm parameters

## Abstract

**Introduction:**

Naturally, in human body, there is a balance between free radicals and the antioxidant system. Yet, cigarette consumption in smokers increases free radicals and decreases antioxidants. A vast body of research showed that the ROS level of seminal fluid is reduced using oral antioxidant complements through enhancing the clearing capacity of free radicals. Thus, the present research aimed to explore the effect of vitamin complement consumption on changing male infertility indices among smokers.

**Methods:**

In the present randomized clinical trial which was single-blinded, 50 infertile oligospermic and asthenospermic men participated. They were selected in a randomized convenient sampling method. Before the study began, a semen sample was taken from all participants for analysis. Subsequently, the patients received 30 mg of Q10 coenzyme, 8 mg of zinc, 100 mg of vitamin C, 12 mg of vitamin E, 400 mg of folic acid for a whole duration of 3 months on a daily basis (once a day) and 200 mg of selenium every other day after lunch. A second sample of seminal fluid was taken from patients and semen parameters were compared between the pre- and post-test. The relevant statistical analyses were conducted in SPSS.23.

**Results:**

A comparison of pre-test and post-test results revealed that all sperm parameters including the volume, morphology, motion, count and progressive motility were significantly increased after the intervention (p ≥ 005). Moreover, a statistically significant improvement was observed in the pH and concentration of seminal fluid.

**Conclusion:**

The present research showed that consuming vitamin complements (30 mg of Q10, mg of zinc, 100 mg of vitamin C, 12 mg of vitamin E, 400 mg of folic acid) once a day as well as 200 mg of selenium every other day can effectively improve the qualitative parameters (pH and concentration) and quantitative parameters (volume, motion, morphology, count and progressive motility) in infertile male smokers.

## 1. Introduction

Infertility implies inability to conceive after at least one year of unprotected intercourse [1]. About 20% of infertility is explained by male factors. Moreover, in 30–40% of other cases of infertility, both male and female factors are involved [2]. Smoking can adversely affect sperm parameters. As shown in a vast body of research, smokers have a lower volume of semen and lower sperm count and motion than non-smokers [3]. Currently, there are more than 10 million smokers in Iran [4]. Smoking can increase free radicals and decrease antioxidants [5]. Naturally, there is a balance between free radicals and antioxidants inside human body. However, if for any reason such as environmental contamination (cigarette consumption), free radicals are more profusely produced or the antioxidant defense system is weakened, it can lead to oxidative stress [6]. Thus, smoking can raise the number of free radicals inside body and hence can induce oxidative stress [7]. A myriad of research has shown that the ROS level of seminal fluid can be lowered using oral antioxidants that help to amplify the clearing capacity of free radicals [8]. In their research, Lingappa et al. (2015) explored the effect of smoking on sperm parameters in B.G. Nagara medical institution. Their research findings revealed that smoking affected sperm motion and count negatively. Yet, this negative effect on the former exceeded that of the latter. The results also showed that the vital parameters of semen such as sperm motion and motility were reduced [9]. Prescription of L-Carnitine, Arginine, zinc, selenium, vitamin B12 as well as many antioxidants such as vitamins C, E and glutathione and Q10 helped to improve sperm parameters [10]. In some research by Kodama et al., oral prescription of vitamin C plus vitamin E and glutathione for 2 months proved to increase the count of sperms [11]. Moreover, several studies showed the positive effects of selenium in a proper dosage on the quality of sperm specially sperm count, motion and morphology. Among these studies was the work of research by Macpherson et al. who reported that selenium could positively affect sperm motion in infertile men [12]. Considering the great number of infertile male smokers in community and due to the very limited number of quantitative studies concerning the effect of oral vitamins on semen parameters in infertile male smokers, the present research intended to explore the effect of oral vitamin complements on changing fertility indices in male smokers of Bandar Abbas.

## 2. Materials and methods

The present research was a single-blinded clinical trial. All procedure in this study were approved by the Ethics Committee of Hormozgan University of Medical Sciences (IR.HUMS.REC.1398.061) and Iranian Registry of Clinical Trial (registration no: IRCT20190617043914N1). The research population comprised infertile oligospermic and asthenospermic men visiting Payambar A'zam public sub-specialized infertility clinic in Bandar Abbas. In the first phase, in advance to the study, all participants were asked for informed consent to take part in the research. This was done through an interview. All patients had at least one year experience of inability to conceive. The inclusion criteria were asthenospermia/oligospermia, cigarette smoking (according to WHO, consuming 100 cigarettes a year during one's life would act as the criterion) [13], more than a year of infertility, no history of surgery in genitals or pelvis such as vasectomy, varicocele, hernia, etc., no alcohol consumption or drug addiction, no chronic physical/mental disease including cardiac, renal, immunological, cancer, hepatic and AIDS, no consumption of vitamin complements within the past 2 months, no exposure to radiation during work or routine activities. The main exclusion criteria were azoospermia or reports of sexual problems, use of any unscheduled vitamin complement, ICSI candidate for intense spermographic disorders and unwillingness to take part in the research. The initial inclusion information was obtained via a checklist developed by the researcher. In the second phase, the subjects were visited by an urologist. Their seminal fluid test results were examined. Eventually, 50 asthenospermic and oligospermic subjects were selected. They were informed of the need for a seminal fluid test so as not to have an intercourse for 3–4 days. Upon arrival at the lab, a sample was taken through masturbation. Once taken, the sample was stored in an incubator at 37 degrees for 20 minutes to liquefy. Computerized seminal fluid analysis was done in vitro based on WHO standards. For all subjects, color, volume, viscosity, count, motion, morphology and pH were analyzed.

Upon receiving the test results, each patient visited the same physician and followed the prescribed medical diet in proper dosage and time. This procedure took 3 months. The prescribed medicine contained 30 mg of Q10, 8 mg of zinc, 100 mg of vitamin C, 12 mg of vitamin E, 400 µg of folic acid once a day and 200 mg of selenium every other day after lunch. To control the complements and prevent attrition, every two weeks the subjects were contacted through phone calls. At the end of the research, the remaining number of tablets or capsules was counted to see the extent to which the subjects followed the prescribed medical diet. Participants who had not taken more than 10% of the tablets were eliminated from the study. At the end of the third month, all subjects were informed to come for a seminal fluid test after 3 days of refraining from sexual intercourse. They referred to the same lab as for the first test. The same full list of information obtained for the first test was again obtained for this second test. For data analysis, the data entered SPSS.23. Mean and standard deviation were used to describe quantitative data. Frequency and percentage were used to describe qualitative data. To test the research hypotheses, a test of normality (Shapiro-Wilk) was run as well as a test of variance equality (Levene's test). One-way ANOVA was run to compare quantitative data. Chi-squared test was used to compare qualitative data. Paired-sample T-test was used to compare the means cores of quantitative data before and after the intervention. The significance level was set at p ≤ 0.05.

## 3. Results

The mean age of the patients was 32.24 ± 6.87 years; the length of their infertility was 4.16 ± 2.82 years. Sperm parameters were divided in two groups of qualitative and quantitative. Each had a certain statistical analysis. The mean scores for sperm parameters can be observed before the intervention and after 3 months of receiving antioxidant vitamins in Table 1.

Quantitative parameters after intervention:

To compare the mean scores of sperm parameters before and after intervention, paired-sample T-test was run, as can be observed in Table 1.

Comparison of mean sperm parameters before and after intervention showed that the mean volume of sperm changed from 3.48 ± 1.44 to 3.71 ± 1.42; sperm motion changed from 27.22 ± 13.69 to 31.85 ± 5.82; sperm morphology changed from 23.22 ± 23.28 to 33.60 ± 20.01; sperm count turned from 21.76 ± 23.02 to 23.22 ± 23.28; progressive motility was increased from 9.82 ± 9.10 to 11.57 ± 10.18.

All sperm parameters including total motility, morphology, count and progressive motility were significantly increased after the intervention (p ≥ 0.005). Yet, pH (in the parametric test) was decreased but not to a statistically significant extent (p = 0.07).

**Table 1.** Comparison of mean scores for sperm parameters before and after intervention.

Sperm Parameter	Group	F.	Mean	Sd	T-Value	P-Value
Volume	Pre-test	50	3.48	1.44	2.20	0.032
	Post-test	50	3.71	1.42		
Ph	Pre-test	50	7.31	0.29	1.85	0.070
	Post-test	50	7.27	0.18		
Total Motility	Pre-test	50	27.22	13.69	3.96	0.001
	Post-test	50	31.85	15.82		
Morphology	Pre-test	50	23.22	23.28	3.14	0.003
	Post-test	50	33.60	20.01		
Count	Pre-test	50	21.76	23.02	4.01	0.001
	Post-test	50	23.22	23.28		
Motility	Pre-test	50	9.82	9.10	5.87	0.001
	Post-test	50	11.57	10.18		

Qualitative parameters after intervention:

To better compare the effectiveness of therapeutic methods in sperm pH, the pH of patients' sperm was classified as either normal or abnormal. pH at or above 7.2 was taken as normal and below that degree was taken as abnormal.

To compare the quality of pH before and after the intervention, McNamara's test was run. The results showed that from among 14 subjects with abnormal pH = 5 (10%) (p = 0.001) had a return to a normal extent of pH. This change was statistically significant and showed a significant improvement.

In terms of sperm concentration, the subjects were divided in 3 groups (below normal, normal and above normal). The test results revealed that 2 of 16 subjects had an above-normal sperm concentration (12.2%) (p = 0.001). Their sperm concentration returned to a normal state which was statistically significant (p = 0.001).

## 4. Discussion

Comparison of patients' sperm parameters before and after the interventions showed that consuming vitamin complements (30 mg of Q10, 8 mg of zinc, 100 mg of vitamin C, 12 mg of vitamin E, 400 µg of folic acid once a day and 200 mg of selenium every other day) can significantly increase sperm volume, total motility, morphology, count and progressive motility. Moreover, sperm pH and concentration were improved. The present research was a single-blinded clinical trial on both semen parameters and the effect of antioxidant vitamin. Thus, there is no other similar research to directly compare the results with. Yet, other studies looked into more details and discussed them accordingly. The present findings are comparable with the research by Zareba et al. (2013). Yet, they were not consistent with these findings. Zareba et al. found no statistically significant correlation between the vitamin-rich (A,E) diet and all sperm parameters [14]. Moreover, Kacem et al. (2014) observed no significant difference in sperm parameters after receiving antioxidant complements [15]. This may be due to the fact that in the study of Kassem et al., Patients in addition to oral antioxidant use also had assisted reproductive methods such as IVF during the study, while in the present study patients who used assisted reproductive methods. Are excluded. However, in the research conducted by Islamian et al. (2012), the combined effect of vitamin D complement and docosahexaenoic acid was investigated on oxidative stress indices of semen in asthenospermic men. The results revealed that the combined treatment with fatty acid complement, docosahexaenoic acid and vitamin E in asthenospermic men managed to reduce oxidative stress in seminal plasma. Consequently, the oral application of antioxidants can potentially be a way to stop oxidative damage to sperm in infertile men [16]. In some other research, Keshavars et al. (2011) explored the antioxidant effects of vitamins C and E on the oxidative stress induced by sulfasalazine in rats' testicles. Sulfasalazine showed to increase oxidative stress and thus disrupted spermatogenesis. Vitamins E, C showed to lower oxidative stress and thus prevented the harmful effects of sulfasalazine on spermatogenesis [17]. Moreover, several studies attested to the positive effect of an appropriate dose of selenium on the quality of sperm viability, motility and morphology. Among them was the work of research by Macpherson et al. who reported that selenium improved sperm total motility in infertile men [12]. In some other research, the effect of Q10 was explored in male infertility. One such study was conducted by Lewin et al. who showed that the oral application of Q10 (60 mg/kg) could improve fertility in the ICSI of normospermic subjects [18]. In their study, Hassanali et al. (2007) showed that zinc in the plasma of seminal fluid was positively correlated with sperm count and negatively correlated with sperm total motility in normospermic and oligospermic men but correlated with sperm morphology [19]. Other works of research can be observed in Table 2.

**Table 2.** Results of consuming antioxidant consumption on semen parameters in the related literature.

Author(s)	Sample size	Target population	Antioxidant	Consumption	Length of consumption	Result(s)
Elsheikh et al. (2015) [20]	90	Oligo-asthenospermia	Vitamin E (400 mg) + clomifene citrate (25 mg)	Daily	6 months	Increased sperm concentration (p = 0.001)
Safarinezhad (2009) [21]	212	Oligozoospermia Asthenospermia	Q10 (300 mg)	Daily	26 weeks	Increased sperm concentration and total motility (p = 0.001)
Nouri et al. (2007) [22]	60	Asthenozoospermia	Vitamin E (400 mg) + vitamin C (1000 mg)	Daily	2 months	Except for increased sperm total motility (p ≤ 0.05), no Significant effect on other parameters
Keskes-Ammar et al. (2003) [23]	72	Infertile male	Vitamin E (400 mg)+selenium (225 mg)	Daily	3 months	Increased sperm total motility and concentration (p ≤ 0.05) and no significant effect on semen morphology (p ≥ 0.05)
Kodama et al. (1997) [11]	14	Infertile male	Vitamin E (20 mg) + vitamin C (200 mg) + glutathione (400 mg)	Daily	2 months	Increased sperm count (p ≤ 0.05)
Suleiman et al. (1996) [24]	52	asthenozoospermia	Infused vitamin E (100 mg)	3 times a day	6 months	Increased motility (p ≤0.05)

## 5. Conclusion

Consuming 30 mg of Q10, 8 mg of zinc, 100 mg of vitamin C, 12 mg of vitamin E, 400 µg of folic acid once a day and 200 mg of selenium every other day showed to positively affect qualitative parameters (concentration and pH) as well as quantitative parameters (volume, total motility, morphology, count and progressive motility). Yet, there is a need for further research with more subjects and research at molecular level.
